# Predicting breast cancer response to neoadjuvant treatment using multi-feature MRI: results from the I-SPY 2 TRIAL

**DOI:** 10.1038/s41523-020-00203-7

**Published:** 2020-11-27

**Authors:** Wen Li, David C. Newitt, Jessica Gibbs, Lisa J. Wilmes, Ella F. Jones, Vignesh A. Arasu, Fredrik Strand, Natsuko Onishi, Alex Anh-Tu Nguyen, John Kornak, Bonnie N. Joe, Elissa R. Price, Haydee Ojeda-Fournier, Mohammad Eghtedari, Kathryn W. Zamora, Stefanie A. Woodard, Heidi Umphrey, Wanda Bernreuter, Michael Nelson, An Ly Church, Patrick Bolan, Theresa Kuritza, Kathleen Ward, Kevin Morley, Dulcy Wolverton, Kelly Fountain, Dan Lopez-Paniagua, Lara Hardesty, Kathy Brandt, Elizabeth S. McDonald, Mark Rosen, Despina Kontos, Hiroyuki Abe, Deepa Sheth, Erin P. Crane, Charlotte Dillis, Pulin Sheth, Linda Hovanessian-Larsen, Dae Hee Bang, Bruce Porter, Karen Y. Oh, Neda Jafarian, Alina Tudorica, Bethany L. Niell, Jennifer Drukteinis, Mary S. Newell, Michael A. Cohen, Marina Giurescu, Elise Berman, Constance Lehman, Savannah C. Partridge, Kimberly A. Fitzpatrick, Marisa H. Borders, Wei T. Yang, Basak Dogan, Sally Goudreau, Thomas Chenevert, Christina Yau, Angela DeMichele, Don Berry, Laura J. Esserman, Nola M. Hylton

**Affiliations:** 1 University of California, San Francisco, CA USA; 2 Karolinska Institute, Stockholm, Sweden; 3 University of California, San Diego, CA USA; 4 University of Alabama, Birmingham, AL USA; 5 University of Minnesota, Minneapolis, MN USA; 6 Loyola University, Maywood, IL USA; 7 University of Colorado, Denver, CO USA; 8 Mayo Clinic, Rochester, NY USA; 9 University of Pennsylvania, Philadelphia, PA USA; 10 University of Chicago, Chicago, IL USA; 11 Georgetown University, Georgetown, DC USA; 12 University of Southern California, Los Angeles, CA USA; 13 Swedish Cancer Institute, Seattle, WA USA; 14 Oregon Health & Science University, Portland, OR USA; 15 Moffitt Cancer Center, Tampa, FL USA; 16 Emory University, Atlanta, GA USA; 17 Mayo Clinic, Scottsdale, AZ USA; 18 Inova Health System, Falls Church, VA USA; 19 University of Washington, Seattle, WA USA; 20 University of Arizona, Tucson, AZ USA; 21 University of Texas, M.D. Anderson Cancer Center, Houston, TX USA; 22 University of Texas Southwestern, Dallas, TX USA; 23 University of Michigan, Ann Arbor, MI USA; 24 Berry Consultants, LLC, Austin, TX USA

**Keywords:** Breast cancer, Cancer imaging

## Abstract

Dynamic contrast-enhanced (DCE) MRI provides both morphological and functional information regarding breast tumor response to neoadjuvant chemotherapy (NAC). The purpose of this retrospective study is to test if prediction models combining multiple MRI features outperform models with single features. Four features were quantitatively calculated in each MRI exam: functional tumor volume, longest diameter, sphericity, and contralateral background parenchymal enhancement. Logistic regression analysis was used to study the relationship between MRI variables and pathologic complete response (pCR). Predictive performance was estimated using the area under the receiver operating characteristic curve (AUC). The full cohort was stratified by hormone receptor (HR) and human epidermal growth factor receptor 2 (HER2) status (positive or negative). A total of 384 patients (median age: 49 y/o) were included. Results showed analysis with combined features achieved higher AUCs than analysis with any feature alone. AUCs estimated for the combined versus highest AUCs among single features were 0.81 (95% confidence interval [CI]: 0.76, 0.86) versus 0.79 (95% CI: 0.73, 0.85) in the full cohort, 0.83 (95% CI: 0.77, 0.92) versus 0.73 (95% CI: 0.61, 0.84) in HR-positive/HER2-negative, 0.88 (95% CI: 0.79, 0.97) versus 0.78 (95% CI: 0.63, 0.89) in HR-positive/HER2-positive, 0.83 (95% CI not available) versus 0.75 (95% CI: 0.46, 0.81) in HR-negative/HER2-positive, and 0.82 (95% CI: 0.74, 0.91) versus 0.75 (95% CI: 0.64, 0.83) in triple negatives. Multi-feature MRI analysis improved pCR prediction over analysis of any individual feature that we examined. Additionally, the improvements in prediction were more notable when analysis was conducted according to cancer subtype.

## Introduction

An important advantage of neoadjuvant chemotherapy (NAC) over adjuvant therapy for locally advanced breast cancer is the ability to monitor treatment response, which allows informed adjustment of the treatment plan. Among imaging methods, magnetic resonance imaging (MRI) is the most accurate for assessing tumor response to NAC^1–5^. Results from the I-SPY 1 TRIAL (CALGB 150007/ACRIN 6657) found that functional tumor volume (FTV) predicted pathologic complete response (pCR) and recurrence-free survival^6,7^. Subsequently, serial measures of FTV during treatment are used in the adaptive randomization engine of the I-SPY 2 trial, designed to accelerate the evaluation of novel agents for breast cancer^8^. Pathologic complete response is the primary endpoint in I-SPY 2.

FTV represents the active portion of tumor volume, as defined by pharmacokinetic thresholds applied to dynamic contrast-enhanced MRI (DCE-MRI)^9^. While FTV has shown effectiveness for the prediction of pCR, there is still potential for improvement, especially in the setting of hormone-positive tumors^10^. Additional features can be derived from the same DCE-MRI data, including longest diameter, sphericity, and contralateral background parenchymal enhancement (BPE). These additional measures have also shown value for prediction of pCR^11–14^. Longest diameter is a standard clinical measurement used to assess tumor response, consistent with the Response Evaluation Criteria in Solid Tumors (RECIST)^15^. Sphericity is a three-dimensional shape feature previously found to be associated with pCR in the I-SPY2 trial^11^. Several studies have shown the association of BPE with breast cancer risk in the screening setting, and decreased BPE has been found to be associated with pCR following neoadjuvant chemotherapy^12–14,16,17^.

This study investigated whether the predictive performance of MRI can be improved over FTV or any single feature alone by using a combination of features measured on DCE-MRI. By providing better prediction of response, MRI can advance personalized treatment and play an important role in assessing whether to change targeted therapies or proceed directly to surgical resection.

## Results

### Patient characteristics

**Fig. 1:**
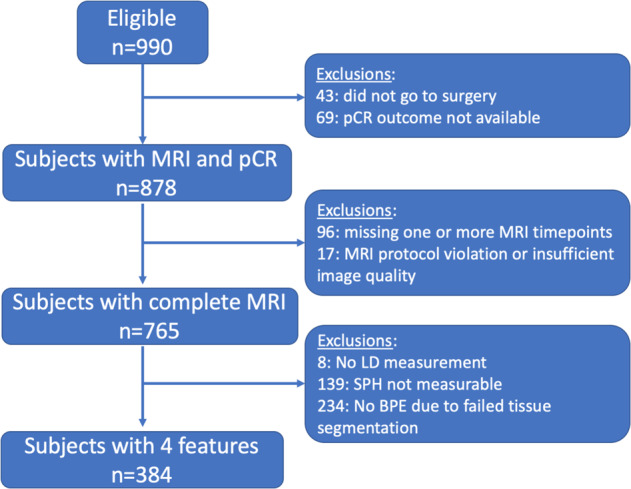
Study subject exclusion criteria. Out of 17 patients excluded for MRI protocol violation or insufficient quality, 10 had protocol violation or technique failure, 6 had obvious motion or were re-positioned after contrast injection, and 1 patient could not tolerate MRI. Image quality issues contributing to the exclusion of BPE values (*n* = 86) were insufficient fat suppression (*n* = 47) or coil inhomogeneity artifact (brightness on the outer edge of the breast, *n* = 37), or both (*n* = 2). The remaining number of exclusions (*n* = 148) were due to the segmentation failure. pCR pathologic complete response, LD longest diameter, SPH sphericity, BPE background parenchymal enhancement.

A total of 384 patients who had complete MRI data and pCR outcome were included in the analysis (see Fig. 1 for patient exclusion details and Table 1 for patient characteristics in the eligible cohort and included cohort). After NAC, 29.7% (114/384) achieved pCR and 70.3% (270/384) did not. The pCR rates in HR/HER2 subgroups were 14.8% (24/162) for HR+/HER2−, 31.7% (19/60) for HR+/HER2+, 66.7% (20/30) for HR−/HER2+, and 38.6% (51/132) for triple negatives (HR−/HER2−). The median age was 49 (interquartile range: 41 to 56, range 24 to 77) years. There was no statistically significant difference (*p* = 0.48) in age between patients eligible (median age: 49; interquartile range: 41 to 56) and analysis (median age: 48.5; interquartile range: 41 to 56). There were no statistically significant differences with respect to race (*p* = 0.54), HR/HER2 subtype (*p* = 0.61), menopausal status (*p* = 0.83), or treatment (*p* = 0.72) between eligible and analysis cohorts. pCR rates in the cohort of subjects with MRI and pCR outcomes (*N* = 878, see Fig. 1) were 34.9% (306/878) for the full cohort, 18.6% (64/344) for HR+/HER2−, 36.6% (49/134) for HR+/HER2+, 69.3% (52/75) for HR−/HER2+, and 43.4% (141/325) for triple negatives. Overall pCR rates were higher in this cohort than in the cohort included in the analysis (*N* = 384).

**Table 1:** Patient characteristics (eligible versus included in the analysis).

	Eligible *N* = 990	Analysis *N* = 384	*p*
Age (median with interquartile range)	49 (41–56)	49 (41–56)	0.48
Race			0.54
White	784 (79.2)	315 (82.0)	
Black or African American	121 (12.2)	34 (8.9)	
Asian	68 (6.9)	27 (7.0)	
American Indian or Alaska Native	4 (0.4)	2 (0.5)	
Native Hawaiian or Pacific Islander	5 (0.5)	3 (0.8)	
Mix	7 (0.7)	3 (0.8)	
HR/HER2 subtype			0.61
HR+/HER2−	380 (38.4)	162 (42.2)	
HR+/HER2+	156 (15.8)	60 (15.6)	
HR−/HER2+	89 (9.0)	30 (7.8)	
HR−/HER2− (triple negative)	363 (36.7)	132 (34.4)	
Menopausal status			0.83
Premenopausal	464 (46.9)	181 (47.1)	
Perimenopausal	33 (3.3)	17 (4.4)	
Postmenopausal	291 (29.4)	113 (29.4)	
Not applicable	134 (13.5)	46 (12.0)	
Unknown	68 (6.9)	27 (7.0)	
Treatment			0.72
Experimental drugs	779 (78.7)	303 (78.9)	
Standard drugs (control)	221 (22.3)	81 (21.1)	

*HR* hormone receptor, *HER2* human epidermal growth factor receptor 2. Note — Unless otherwise specified, data in columns 2 and 3 are number of patients, with percentages in parentheses.

### Predict pCR using MRI features

**Fig. 2:**
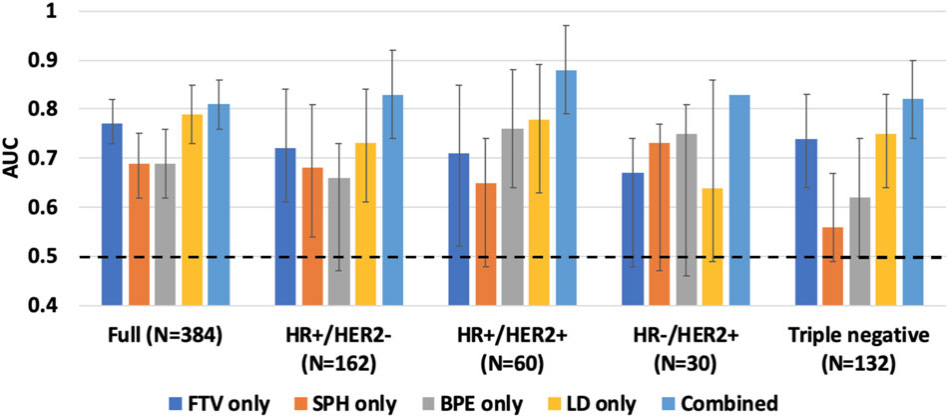
Bar chart of area under the receiver operating characteristic curves (AUCs) for predicting pathologic complete response using single versus combined MRI features. Each column represents an AUC value estimated for the logistic regression model using a single or combined MRI features. MRI features include functional tumor volume (FTV), sphericity (SPH), background parenchymal enhancement (BPE), and longest diameter (LD). AUCs were plotted in the full cohort and in sub-cohorts defined by hormone receptor (HR) and human epidermal growth factor 2 (HER2) status. The error bar shows the 95% confidence interval of each estimated AUC. The black dotted line shows where AUC = 0.5 is.

**Fig. 3:**
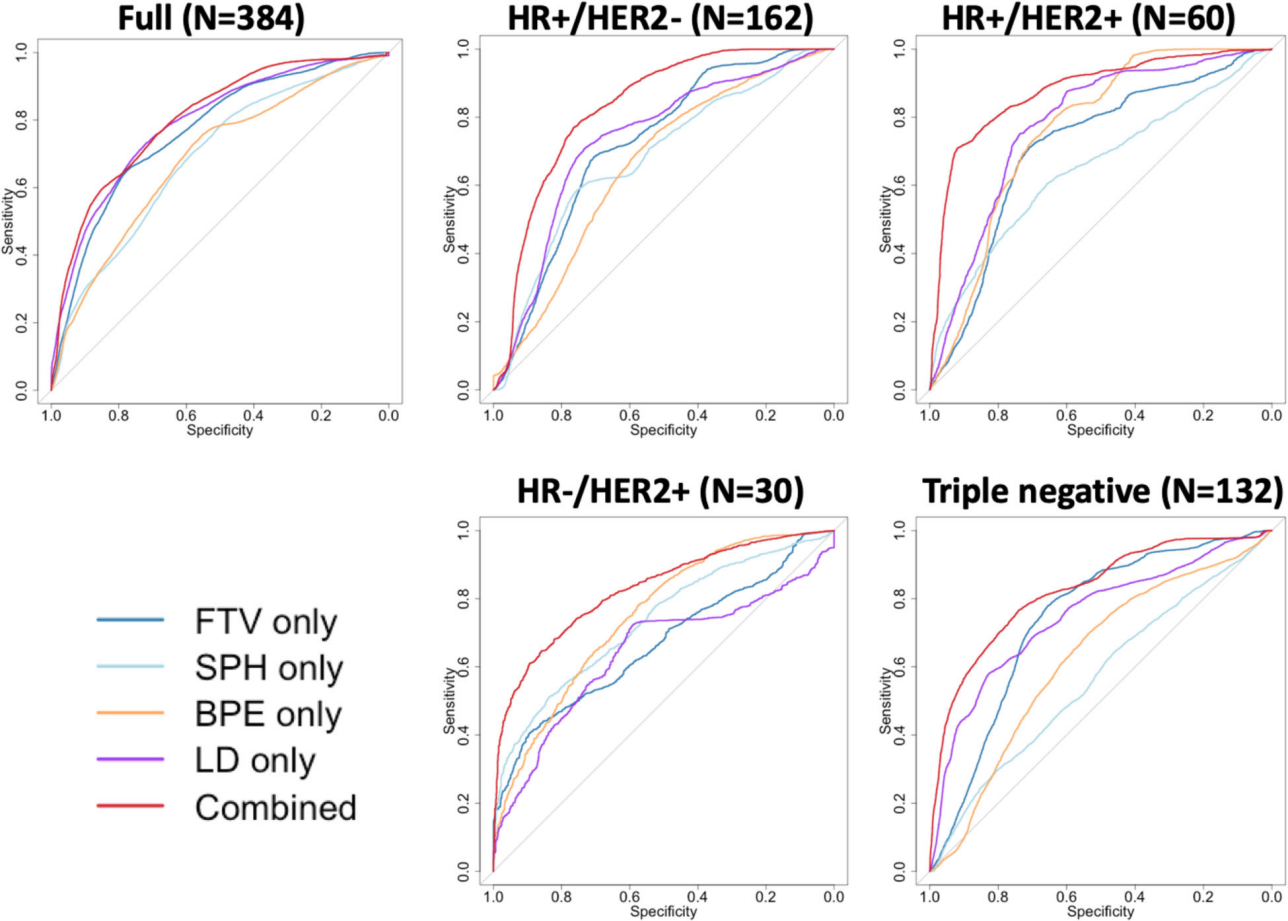
Plots of receiver operating characteristic curves (ROCs) for single versus combination of MRI features. The corresponding areas under the ROC curve (AUCs) are listed in Table 2. MRI features include functional tumor volume (FTV), sphericity (SPH), background parenchymal enhancement (BPE), and longest diameter (LD). ROCs were plotted in the full cohort and in sub-cohorts defined by hormone receptor (HR) and human epidermal growth factor 2 (HER2) status.

Table 2 shows the estimated AUCs (and 95% CIs) for optimized models generated by individual and combined features. Variables included in each model are listed in Supplementary Table 1. Fig. 2 shows the bar charts for visual comparison and Fig. 3 shows the corresponding ROC curves for each AUC value.

**Table 2:** AUCs of optimized models using individual versus combined MRI features.

Model type	Full *N* = 384 pCR rate = 29.7%	HR+/HER2-*N* = 162 pCR rate = 14.8%	HR+/HER2+*N* = 60 pCR rate = 31.7%	HR-/HER2+*N* = 30 pCR rate = 66.7%	HR-/HER2-*N* = 132 pCR rate = 38.6%
FTV only	0.77 (0.73, 0.83)	0.72 (0.61, 0.84)	0.71 (0.52, 0.85)	0.67 (0.48, 0.74)	0.74 (0.64, 0.83)
BPE only	0.69 (0.62, 0.76)	0.66 (0.47, 0.73)	0.76 (0.64, 0.88)	0.75 (0.46, 0.81)	0.62 (0.50, 0.74)
SPH only	0.69 (0.62, 0.75)	0.68 (0.54, 0.81)	0.65 (0.48, 0.74)	0.73 (0.47, 0.77)	0.56 (0.49, 0.67)
LD only	0.79 (0.73, 0.85)	0.73 (0.61, 0.84)	0.78 (0.63, 0.89)	0.64 (0.49, 0.86)	0.75 (0.64, 0.83)
Combined	0.81 (0.76, 0.86)	0.83 (0.77, 0.92)	0.88 (0.79, 0.97)	0.83	0.82 (0.74, 0.91)

Note —Numbers in parentheses are 95% confidence intervals.

Combining multiple MRI features resulted in higher AUC compared to single features alone, in the full cohort and in each breast cancer subtype. In the full cohort, AUC for the combined model was 0.81 (95% CI: 0.76–0.86), which exceeded the highest AUC achieved using a single feature model (LD) at 0.79 (95% CI: 0.73–0.85). The *p*-value of the difference between the two AUCs was <0.001.

Using the combined model within individual subtypes resulted in improved predictive value: an AUC of 0.83 (95% CI: 0.77–0.92, *p* < 0.001) was achieved in HR+/HER2−, 0.88 (95% CI: 0.79–0.97, *p* < 0.001) in HR+/HER2+, and 0.82 (95% CI: 0.74–0.91, *p* < 0.001) in HR−/HER2− (TN). We could not calculate a reliable 95% confidence interval for the AUC of combined features in the HR− /HER2+ subgroup because the number of outcomes was too small (*n* = 20 pCRs; *n* = 10 non-pCRs).

Although AUCs of the combined features were higher than those of individual measures in the full cohort and in subtype cohorts (*p* < 0.001), Fig. 3 shows their relationship on the full scale of sensitivity and specificity. The ROC curves of the combined predictors had greater separation from the ROCs of a single type of predictor for the subtype cohorts than the full cohort.

## Discussion

Given its robust correlation with long-term outcomes, pCR has increasingly become the clinical goal of NAC in locally advanced breast cancer. The ability to use non-invasive methods to accurately predict pCR early in the course of treatment has enormous clinical implications as it would permit personalized, evidence-based escalation or de-escalation of therapy. Our results showed that MRI functional tumor volume-based prediction of pathologic outcome following NAC can be improved using a combination of multiple features, as compared to a single feature alone. Importantly, each of these features can be measured from the same DCE-MRI dataset, requiring no additional image acquisitions.

In support of our findings, previous studies using combined MRI parameters have typically shown higher predictive performance for pCR compared to those using a single parameter. For example, Lee et al compared the ability of pre-treatment DCE-MRI perfusion imaging parameters to predict pCR in 74 breast cancer patients who were treated with NAC followed by surgery^18^. Their retrospective study concluded that the model combining perfusion parameters of contralateral breast background parenchyma and those of the tumor had higher predictive value than each single-parameter model. This also agrees with results published by Hylton et al, who performed a multivariable analysis of the DCE-MRI examinations of 162 women with breast tumors 3 cm or larger^6^, showing that a model combining MRI parameters (longest diameter, functional tumor volume, signal enhancement ratio) and clinical tumor size achieved the highest predictive accuracy for pCR.

Based on our study of HR/HER2 subtype, the improvement in predicting pCR by multi-feature MRI was more notable in individual subtypes than in the full cohort. More interestingly, imaging predictors included in the optimized model were different among subtypes, which indicates that some features may capture the treatment response better than others, depending upon the cancer subtype. For example, studies have shown that tumor sizes measured using MRI were less accurate in HER2+ compared to HER2− subtypes^19,20^. However, the decrease in BPE before and after NAC showed its association with pCR in HER2+ breast cancer^21,22^. Our study showed consistent results as FTV or LD yielded lower AUCs than SPH or BPE in the HR−/HER2+ subtype, where combining them into the prediction model can help improve the predictive performance.

Four MRI features were included in this analysis. They were chosen by having demonstrated clinical relevance. However, there could be many other imaging features in MRI that could also potentially be predictive of pCR. With the advancement of computational technology, radiomics can extract a large number of features and machine-learning algorithms can be used to select biologically or physiologically meaningful features to predict cancer treatment outcomes. In our future studies, other radiomics features will be explored.

Among the four MRI features that we studied, FTV is an IDE-approved algorithm and a well-established imaging biomarker in the I-SPY 1 and 2 trials. Other features all have pitfalls and challenges. LD is a standardized and internationally recognized measurement reported in the ACR Breast Imaging Reporting and Data System (BI-RADS)^23^. However, LD can be subjective and may not capture the functional or physiological changes from treatment. In this study, BPE was calculated fully automatically and therefore avoided reader subjectivity. However, achieving a reliable and automated quantitative BPE measurement is still a challenge. Approximately 30% of the MRI examinations were excluded because of inadequate fibroglandular tissue segmentations. A more reliable quantitative BPE measurement in combination with higher overall image quality standards is needed. SPH is a morphologic measurement of tumor shape. According to its definition, a solid round-shaped tumor has a larger SPH than a diffuse tumor. However, SPH does not accurately differentiate tumor necrosis and multi-centric tumors. In addition, SPH is not measurable when tumor volume has reduced to a minimal residual. We observed better predictive performance by combining these features together than using any single feature alone, which indicates that deficiencies in the individual features may compensate for each other in the prediction of treatment response.

Our study has several limitations. First, all DCE-MRI data in I-SPY 2 were under well-managed assessment and control, but we still observed various quality issues such as different signal-to-noise ratios and insufficient fat suppression. These variations could affect the variability of our MRI feature measurements. Second, SPH was not calculable when FTV was close to zero. This limitation can cause the exclusion of good responders in our analysis. Third, even though we had the advantage of a large sample size for our study (*n* = 384), the patient population was not evenly distributed among cancer subtypes. In particular, the HR−/HER2+ subset had only 30 patients with 10 non-pCRs, which prohibited us from achieving a reliable 95% CI confidence interval for the AUC in this sub-cohort. Fourth, because multiple agents were tested simultaneously in I-SPY 2, patients with the same HR/HER2 status could have received different agents and responded differently. In future analyses, drug efficacy should also be estimated as an independent variable in the prediction model when a larger sample size is available.

In conclusion, our study showed that MRI can provide quantitative information about tumor characteristics, and multi-feature analysis yielded better prediction of pathologic complete response than sole analysis of any of the single features we examined. The improvement in the predictive performance was more notable when analysis was conducted into cancer subtype. Continued work to improve the reliability and predictive performance of individual features is currently underway and further testing of the multi-feature model will be done in expanded I-SPY 2 cohorts.

## Methods

### Patient population

**Fig. 4:**
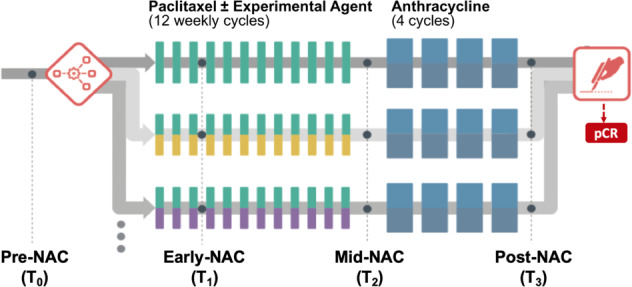
I-SPY 2 study schema and adaptive randomization. Patients were randomized to the standard (paclitaxel for human epidermal growth factor 2 [HER2]-negative or paclitaxel plus trastuzumab for HER2-positive) or one of the experimental drug arms. Participants received a weekly dose of paclitaxel alone (standard) or in combination with an experimental agent for 12 weekly cycles followed by four (every 2–3 weeks) cycles of anthracycline-cyclophosphamide (AC) prior to surgery. MRI examinations were performed at pre-neoadjuvant chemotherapy (NAC) (T0), early NAC (T1), mid-NAC (T2), and post-NAC (T3).

Women 18 years of age and older and diagnosed with locally advanced breast cancer (stage II or III, tumor ≥ 2.5 cm) are eligible to enroll in the I-SPY2 trial (clinical trial number: NCT01042379; registration date: January 5, 2010)^24,25^. A total of 990 patients enrolled in I-SPY 2 from May 2010 to November 2016 and randomized to one of nine completed experimental drug arms or standard of care were considered in this retrospective study. Participants received 12 weekly cycles of paclitaxel alone (standard of care) or in combination with one of nine experimental agents, followed by four cycles of anthracycline-cyclophosphamide (AC) every 2–3 weeks, prior to definitive surgery (Fig. 4)^10^. Patients with HER2-positive cancer also received trastuzumab for the first 12 cycles. In some experimental drug arms, the experimental agent may substitute for one of the standard therapies (paclitaxel or trastuzumab). All participating sites received approval from their institutional review board. All patients provided written informed consent to participate in the study. Subsets of the patient cohort were included in previous studies^10,26,27^.

### MRI acquisition and feature analysis

For each participant, MRI examinations occurred at four sequential time points: pre-treatment (T_0_, pre-NAC), after 3 cycles (T_1_, early NAC), after 12 cycles and between drug regimens (T_2_, mid-NAC), and before surgery (T_3_, post-NAC). All MRI examinations used DCE-MRI, performed according to the predefined I-SPY 2 MRI protocol (described in Supplementary Table 2).

For each DCE-MRI examination, four features were assessed: functional tumor volume (FTV), sphericity (SPH), contralateral background parenchymal enhancement (BPE), and longest diameter (LD). FTV, SPH, and BPE were calculated using in-house software tools developed in the IDL software environment (Exelis Visual Information Solutions, Boulder, Colorado). The FTV method was subsequently replicated on a commercial platform that gained FDA IDE approval in 2010 for use in I-SPY 2^9,28^. LD was measured by the site radiologist and abstracted from clinical MRI reports by study coordinators at each site. Study coordinators, radiologists, and imaging scientists who worked on generating these features were blind to pathologic outcomes.

FTV and SPH were calculated within a 3D volume-of-interest (VOI) defined by the site radiologist or trained imaging coordinator. Early percent enhancement (PE) and signal enhancement ratio (SER) maps were derived by $PE = \frac{{S_1 - S_0}}{{S_0}} \times 100\%$ and $SER = \frac{{S_1 - S_0}}{{S_2 - S_0}}$, where *S*_0_, *S*_1_, and *S*_2_ are signal intensities at pre-contrast, early (approximately 2.5 minutes), and late (approximately 7.5 minutes) post contrast, respectively. FTV was calculated by summing voxel volumes with PE ≥ 70% and SER ≥ 0. As previously described, a threshold different from 70% was applied for a small number of patients when necessary to account for variability in MRI systems and tumor enhancement pattern^9^. In these cases, adjusted thresholds defined at baseline were kept constant for all subsequent MRI examinations. SPH was defined as $\frac{{SA_0}}{{SA_{tumor}}}$, where SA_tumor_ is the surface area of the 3D FTV tumor mask and SA_0_ is the surface area of a perfect sphere of the same volume. Tumor surface area was calculated using a surface meshing analysis. SPH values range from 0 to 1.0, with 1.0 representing a perfect sphere.

BPE was defined as the mean PE of fibroglandular tissue in the contralateral breast. An automated segmentation algorithm was used to identify breast tissue boundaries and a fuzzy c-means clustering algorithm was applied to classify fibroglandular tissue from the segmented breast^29^. BPE was calculated by automatically averaging over the tissue in five continuous axial slices geometrically centered in the superior–inferior direction to characterize tissue in the center of the breast. Illustrations of measuring FTV, LD, SPH, and BPE are shown in Supplementary Fig. 1.

### Pathologic outcome

pCR was defined as the absence of residual invasive disease in the breast and axillary lymph nodes after NAC, measured at surgery. Histopathologic analysis was performed by site pathologists.

### Statistical analysis

Baseline values and percentage changes from baseline were computed for each feature and treated as independent variables in the logistic regression model using binary pCR outcome (1: pCR; 0: non-pCR) as the dependent variable. The area under the curve (AUC) for the receiver operating characteristic (ROC) was used to assess the predictive performance, with 100 repeated 5-fold cross-validation applied to avoid biased estimates of classification accuracy. The 95% confidence interval (CI) of cross-validated AUC was estimated using 1,000 bootstrap resamples. *P*-values of variables in the logistic regression model were estimated by the likelihood-ratio chi-squared test of nested models—with and without the variable being tested. This retrospective analysis was restricted to patients with all four MRI features available at all treatment time points.

Logistic regression models were built using single versus combined MRI features. For single-feature (i.e., FTV, SPH, BPE, or LD) analysis, optimized models were built by selecting variables—from baseline measure and percentage change at T1, T2, T3 compared to the baseline—as independent variables in the logistic regression analysis, and by achieving the highest cross-validated AUCs as mentioned above. For the combined method, all variables from four MRI features available at all treatment time points up to T3 were subject to the variable selection. For single and combined analyses, optimized models were created separately in the full patient cohort and in each of the four breast cancer subtypes defined by HR/HER2 status. Subtype was added as an additional independent categorical variable in the regression model for the full cohort.

The Wilcoxon rank and Fisher’s exact test was used to assess differences by age, HR/HER2 subtype, race, menopausal status at the start of NAC, and treatment (experimental versus standard chemotherapy). AUCs of ROC curves were compared by bootstrapping with 2,000 replicates using a two-sided test.

Statistical analyses were performed using *R* version 3.4.1 (R Foundation for Statistical Computing, Vienna, Austria), where the ‘caret’ package was used for logistic regression analyses^30^, the ‘pROC’ package for ROC analyses^31^, and the ‘boot’ package for calculating 95% CIs for cross-validated AUCs^32,33^. All tests were considered nominally statistically significant when *p* < 0.05.

### Reporting summary

Further information on research design is available in the Nature Research Reporting Summary linked to this article.

## Supplementary information

Supplemental material

Reporting Summary Checklist

## Data Availability

The data generated and analyzed during this study are described in the following data record: 10.6084/m9.figshare.12912191^34^. The datasets are as follows: the original acquired and derived MRI DICOM data, under the title “I-SPY 2 MRI Collection”, and an Excel file called “Multi-feature MRI NACT Data.xlsx”. These will be deposited and be publicly available in NCI The Cancer Imaging Archive (TCIA): https://www.cancerimagingarchive.net/. However, due to technical limitations with the deposition and curation of the data, their release date is anticipated to be late 2020. When they become available, this metadata record associated with this article^34^ will be updated to version 2 to link the TCIA data DOI. In the meantime, please contact the corresponding author with data queries.
