# Supplementary material for: Predicting breast cancer response to neoadjuvant treatment using multi-feature MRI: results from the I-SPY 2 TRIAL
Source: NPJ Breast Cancer. 2020 Nov 27;6:63. doi: 10.1038/s41523-020-00203-7 (PMC7695723; doi:10.1038/s41523-020-00203-7)
Supplement: Supplementary file 1 — Supplemental material [file 41523_2020_203_MOESM1_ESM.pdf]

Supplemental

Supplementary Table 1. Variables included in optimized models

**Model type**	**Included variables**
**All subtypes (N=384, pCR rate = 114/384, 29.7%)**	
FTV only	**Subtype**, **%ΔFTV0_1**, **%ΔFTV0_2**
SPH only	**Subtype**, **SPH0**
BPE only	**Subtype**, BPE0, %ΔBPE0_1, %ΔBPE0_3
LD only	**Subtype**, **LD0**, **%ΔLD0_2**, **%ΔLD0_3**
Combined	**Subtype**, **%ΔFTV0_2**, **%ΔSPH0_2**, **BPE0**, **%ΔLD0_2**, **%ΔLD0_3**
**HR+/HER2- (N=162, pCR rate = 24/162, 14.8%)**	
FTV only	%ΔFTV0_1, %ΔFTV0_2, %ΔFTV0_3
SPH only	**%ΔSPH0_1**, %ΔSPH0_2
BPE only	BPE0, %ΔBPE0_1, %ΔBPE0_3
LD only	%ΔLD0_2, **%ΔLD0_3**
Combined	%ΔFTV0_1, **%ΔFTV0_3**, **%ΔSPH0_1**, BPE0, %ΔBPE0_2, **LD0**, **%ΔLD0_2**
**HR+/HER2+ (N=60, pCR rate = 19/60, 31.7%)**	
FTV only	%ΔFTV0_1, %ΔFTV0_3
SPH only	%ΔSPH0_1
BPE only	**%ΔBPE0_3**
LD only	%ΔLD0_1, **%ΔLD0_3**
Combined	**%ΔSPH0_2**, **%ΔBPE0_3**, **%ΔLD0_3**
**HR-/HER2+ (N=30, pCR rate = 20/30, 66.7%)**	
FTV only	%ΔFTV0_3
SPH only	SPH0, %ΔSPH0_1
BPE only	BPE0, %ΔBPE0_1, %ΔBPE0_2
LD only	LD0, %ΔLD0_2
Combined	%ΔFTV0_3, SPH0, %ΔSPH0_1, %ΔBPE0_1
**HR-/HER2- (triple negative, N=132, pCR rate = 51/132, 38.6%)**	
FTV only	%ΔFTV0_1, **%ΔFTV0_2**
SPH only	%ΔSPH0_1
BPE only	**BPE0**, %ΔBPE0_2
LD only	**%ΔLD0_2**
Combined	**%ΔFTV0_2**, **%ΔSPH0_2**, **BPE0**, **%ΔBPE0_2**, **%ΔLD0_2**

*bolded AUCs were the highest AUC in each cohort. Bolded variables were with $p<0.05$.

Supplementary Table 2. Standardized I-SPY 2 DCE-MRI acquisition parameters

Parameter	Value
Sequence type	Gradient echo (GE)
2D or 3D sequence	3D
Slice orientation	Axial
Laterality	Bilateral
Frequency direction	A/P
Phase direction	R/L
FOV - frequency	260 – 360 mm
FOV - phase	260 – 360 mm
Matrix – frequency (acquired)	384 – 512
In-plane resolution	≤ 1.4 mm
Fat-suppression	Active fat-sat recommended
TR	4 – 10 ms
TE	Minimum TE
Flit angle	10 – 20 degrees
B values	N/A
Slice thickness (acquired, not interpoloated)	≤ 2.5 mm
Number of slices	≥ 60; complete bilateral coverage
Slice Gap	No gap
Parallel imaging factor	≤ 2
No. of excitations or averages	≤ 2
k-space ordering	-k to +k (standard, non-centric)
Sequence acquisition time	80 sec ≤ scan time ≤ 100 sec
Total post-contrast imaging duration	≥ 8 minutes following injection

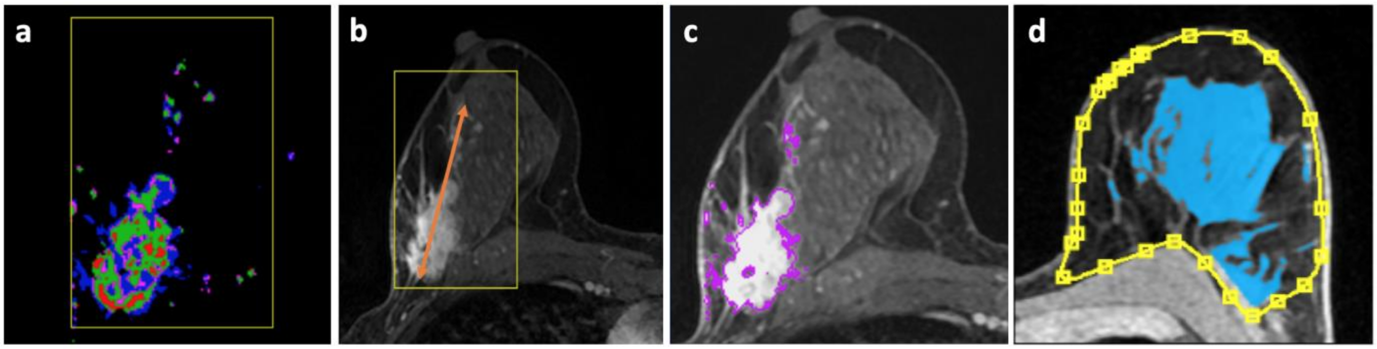

Supplementary Figure 1. Illustration of MRI feature measurements. A. functional tumor volume (FTV). Voxels with percentage enhancement (PE) ≥ 70% within the volume-of-interest shown as the yellow rectangular frame are counted in the FTV calculation. Color represents different levels of signal enhancement ratio (red: SER > 1.1; green: 0.9 ≤ SER ≤ 1.1; blue: SER < 0.9). B. longest diameter (LD). C. sphericity (SPH). Voxels in color mark where tumor boundary is. D. background parenchymal enhancement (BPE). Yellow curve shows the whole breast segmentation and voxels in blue shows the segmented fibroglandular tissue within the breast. BPE is calculated as the average PE among all blue voxels.
