# Supplementary material for: Predicting breast cancer response to neoadjuvant treatment using multi-feature MRI: results from the I-SPY 2 TRIAL
Source: NPJ Breast Cancer. 2020 Nov 27;6:63. doi: 10.1038/s41523-020-00203-7 (PMC7695723; doi:10.1038/s41523-020-00203-7)
Supplement: Supplementary file 2 — Reporting Summary Checklist [file 41523_2020_203_MOESM2_ESM.pdf]

nature research

Corresponding author(s): Nola M. Hylton

Last updated by author(s): Oct 14, 2020

Reporting Summary

Nature Research wishes to improve the reproducibility of the work that we publish. This form provides structure for consistency and transparency in reporting. For further information on Nature Research policies, see our Editorial Policies and the Editorial Policy Checklist.

Statistics

For all statistical analyses, confirm that the following items are present in the figure legend, table legend, main text, or Methods section.

n/a	Confirmed	
☐	☒	The exact sample size (*n*) for each experimental group/condition, given as a discrete number and unit of measurement
☐	☒	A statement on whether measurements were taken from distinct samples or whether the same sample was measured repeatedly
☐	☒	The statistical test(s) used AND whether they are one- or two-sided<br>*Only common tests should be described solely by name; describe more complex techniques in the Methods section.*
☐	☒	A description of all covariates tested
☒	☐	A description of any assumptions or corrections, such as tests of normality and adjustment for multiple comparisons
☐	☒	A full description of the statistical parameters including central tendency (e.g. means) or other basic estimates (e.g. regression coefficient) AND variation (e.g. standard deviation) or associated estimates of uncertainty (e.g. confidence intervals)
☐	☒	For null hypothesis testing, the test statistic (e.g. *F*, *t*, *r*) with confidence intervals, effect sizes, degrees of freedom and *P* value noted<br>*Give P values as exact values whenever suitable.*
☒	☐	For Bayesian analysis, information on the choice of priors and Markov chain Monte Carlo settings
☒	☐	For hierarchical and complex designs, identification of the appropriate level for tests and full reporting of outcomes
☒	☐	Estimates of effect sizes (e.g. Cohen's *d*, Pearson's *r*), indicating how they were calculated

Our web collection on statistics for biologists contains articles on many of the points above.

Software and code

Policy information about availability of computer code

Data collection	Our MRI data were collected from MRI scanners at multiple centers and scanners were from multiple vendors.
Data analysis	Image feature measurements were done using in-house software BRTOOL developed with IDL (Exelis Visual Information Solutions, Boulder, Colorado). All statistical analysis were done using R (version 3.4.1).

For manuscripts utilizing custom algorithms or software that are central to the research but not yet described in published literature, software must be made available to editors and reviewers. We strongly encourage code deposition in a community repository (e.g. GitHub). See the Nature Research guidelines for submitting code & software for further information.

Data

Policy information about availability of data

All manuscripts must include a data availability statement. This statement should provide the following information, where applicable:

- Accession codes, unique identifiers, or web links for publicly available datasets
- A list of figures that have associated raw data
- A description of any restrictions on data availability

Data are available from the authors upon reasonable request and with permission of I-SPY 2 DAPC (Data Access and Publications Committee).

Field-specific reporting

Please select the one below that is the best fit for your research. If you are not sure, read the appropriate sections before making your selection.

☒ Life sciences ☐ Behavioural & social sciences ☐ Ecological, evolutionary & environmental sciences

For a reference copy of the document with all sections, see nature.com/documents/nr-reporting-summary-flat.pdf

Life sciences study design

All studies must disclose on these points even when the disclosure is negative.

Sample size	We chose the sample size because it was the data available at the time of analysis.
Data exclusions	We provide a detailed diagram in the manuscript on how and why some data were excluded. Patients without pathological outcome and missing MRI exams were excluded. Patients with insufficient image quality or unmeasurable imaging features were also excluded.
Replication	We performed 5-fold cross validation to estimate AUCs for logistic regression models with multiple variables to avoid over-fitting. And the confidence interval of the AUC was estimated by bootstrapping to ensure the generalizability of the study. However, we could not find a reliable confidence interval for HR-/HER2+ cancer subtype which had the smallest cohort when a large number of variables were tested in the model.
Randomization	Although the data we analyzed were from a randomization clinical trial, we did not perform a experimental vs. control study.
Blinding	Authors who performed imaging analysis were all blinded to patients treatment/subtype/outcome.

Reporting for specific materials, systems and methods

We require information from authors about some types of materials, experimental systems and methods used in many studies. Here, indicate whether each material, system or method listed is relevant to your study. If you are not sure if a list item applies to your research, read the appropriate section before selecting a response.

Materials & experimental systems

n/a	Involved in the study	
☒	☐	Antibodies
☒	☐	Eukaryotic cell lines
☒	☐	Palaeontology and archaeology
☒	☐	Animals and other organisms
☐	☒	Human research participants
☒	☐	Clinical data
☒	☐	Dual use research of concern

Methods

n/a	Involved in the study	
☒	☐	ChIP-seq
☒	☐	Flow cytometry
☒	☐	MRI-based neuroimaging

Human research participants

Policy information about studies involving human research participants

Population characteristics	This is a sub-cohort of participants in I-SPY 2 TRIAL. Median age is 49, range 23 -- 77 years old. Women in this cohort were diagnosed with stage II or III invasive breast cancer with tumor size >= 2.5 cm. All patients underwent neoadjuvant chemotherapy for breast cancer.
Recruitment	Patients recruited by I-SPY. since patients were already screened to have high risk breast cancer (by MammaPrint) and tumor size >= 2.5 cm, there is a possibility that some features used in this study such as tumor volume, longest diameter, or sphericity will not be measurable on small tumors.
Ethics oversight	University of California, San Francisco; University of California, San Diego; University of Alabama; University of Minnesota; Loyola University; University of Colorado, Denver; Mayo Clinic, Rochester; University of Pennsylvania; University of Chicago; Georgetown University; University of Southern California; Swedish Cancer Institute; Oregon Health & Science University; Moffitt Cancer Center; Emory University; Mayo Clinic, Scottsdale; Inova Health System; University of Washington; University of Arizona; University of Texas, M.D. Anderson Cancer Center; University of Texas Southwestern; University of Michigan

Note that full information on the approval of the study protocol must also be provided in the manuscript.
